# Supplementary material for: Understanding the perspectives of people with dementia and family carers about clinical pharmacists in primary care: A qualitative study
Source: PLoS One. 2025 Aug 13;20(8):e0330028. doi: 10.1371/journal.pone.0330028 (PMC12348983; doi:10.1371/journal.pone.0330028)
Supplement: S1 Appendix — (DOCX) [file pone.0330028.s001.docx]

**Appendix 1**

**DCPharm Topic Guide - Carers**

**Introduction**

- Thank participant for taking part, introduce self.
- Briefly explain purpose of research: To understand your experience of working with clinical pharmacists and how they help you, and the person you are caring for.
- Recap consent form (if completed separate to interview):
- Participation is voluntary – can stop interview at any time or choose not to answer questions.
- Withdrawing data – up to six weeks after interview
- Confidentiality – unless risk of harm to you or others.
- Anonymity – personal details not shared, not identifiable in reports/publications.
- Recording – for quotes, and to check accuracy.
- Length of interview: up to 60 minutes

1. Can you begin by telling me how long you have been caring for [name of person they care for]?

(probe about relationship to them)

1. So, you are here because you have experience in your caring role of seeing or talking to a pharmacist that works in a GP practice. You may have heard them being called a clinical pharmacist. What do you understand about the role of pharmacists that are based in GP practices and what they do? (prompt if needed):
   1. Able to offer specialist/focus advice and care?
   2. More in-depth knowledge of dementia and/or medicines?
2. Could you tell me a little bit about how you have worked with your clinical pharmacist to support you and (name of person they care for)?
   1. How did you find out about being able to access a clinical pharmacist? (did they contact you/was referred)
   2. How accessible it was (Only during GP hours, or less, direct phone/practice switchboard/email)
   3. What did they specifically help with? (i.e. specifically to do with dementia diagnosis for person you care for?)
   4. Did they help with anything other than dementia? (if so, what?)
   5. Have they helped you with communicating with the GP about any particular requests? (Can you give an example e.g. support with a prescription, coming off a medication, asking for a different method of administration?)
3. Could you tell me what medication or why (name of person they cared for) was taking medication?
   1. Did a clinical pharmacist do a review of this medication? (If yes, what happened in the review, what did you talk about?)
   2. Have there been any changes to these medications? (If yes, why? Who was involved in changing them?)
   3. Did any medication get stopped? (If yes, why was it stopped)
   4. Did the clinical pharmacist discuss risks to medication in the review, as well as any benefits?
4. What would you say have been the main ways in which a clinical pharmacist has supported or helped you and the person you are caring for (or not)?
   1. Examples specifically to do with your role as a carer/living with dementia?
   2. New medications/planning medications/changes to medication or coming off meds?
   3. Understanding side effects to medication
   4. Understanding why medications are prescribed and what they do?
   5. Help with ideas around swallowing – i.e. changing the type of medicines
   6. During discharge from hospital back to GP?
   7. Accessing secondary care or help with community referrals e.g. social prescribing?
   8. Examples of support that has not focused on medication e.g. help with social isolation, digital exclusion, issues within home environment?
5. How did the clinical pharmacist interact and include (name of person with dementia) in the appointments?
   1. Any particular strategies to involve them in any decisions for medication changes? Any examples of this?
   2. How was the communication in appointments? E.g. offered face-to-face appointments to aid communication?
   3. Was a rapport built between them?
6. Can you tell me how your experience of seeing a clinical pharmacist has differed to a seeing another pharmacist in your local pharmacy?
   1. Level/amount of advice or information give?
   2. Type of support - more specialist/tailored to needs of patient?
   3. Process
   4. Outcome
   5. Relationship i.e. able to build rapport, friendly, trust…
7. How do you think the services provided by these pharmacists could be improved to specifically support people living with dementia and family carers like yourself?
   1. Seeing a clinical pharmacist more often? (how might this help?)
   2. Understanding needs of culturally diverse clients (behaviour/language barriers)
   3. More flexibility i.e. appointment times, home visits, length of appointment...
   4. Improved accessibility (e.g., direct phone number/practice switchboard/email, face-to-face vs. remote access?)
   5. Clearer communication during appointment?
8. What would you tell a clinical pharmacist about the specific support that someone with dementia and their carer needs?

a. Do you have an example of when you have done this?

1. What kind of advice would you offer to another family carer or person living with dementia if they were unsure about seeing a clinical pharmacist?

a. Do you have an example of when you have done this?

1. In an ideal word, what three things would you like to change or improve about clinical pharmacists in general?
   1. Want more of them?
   2. More resources to do their job/support you better? If so what does that look like?
   3. What would be most helpful or beneficial to you/people with dementia in the future?
   4. Is there anything that you think could help pharmacists directly?
   5. Any thoughts on priorities for future research? (only ask if feel this will be understood)

End of interview follow up questions

- Do you feel you could cope with the length of the interview?
- Did you find talking in the interview helpful?
- Did you feel the interview caused you distress?

**DCPharm Topic Guide: People living with dementia**

**Introduction**

- Thank participant for taking part, introduce self.
- Briefly explain purpose of research: To learn about your experiences of support from healthcare services particularly about your use of pharmacy/chemist services.
- Recap consent form (if completed separate to interview):
- Participation is voluntary – can stop interview at any time or choose not to answer questions.
- Withdrawing data – up to six weeks after interview
- Confidentiality – unless risk of harm to you or others.
- Anonymity – personal details not shared, not identifiable in reports/publications.
- Recording – for quotes, and to check accuracy.
- Length of interview: up to 30 minutes.

1. Can you begin by telling me a little bit more about your yourself and your health?
2. You mentioned dementia (or equivalent) … can you tell me a bit about that. Recap on type of dementia (if unclear) and how long ago diagnosed – if known or appropriate.
3. How are you managing day-to-day?
   1. E.g. around the house, looking after yourself?
   2. Is there anything you struggle with day-to-day because of your health?
   3. Do you get any paid help from anyone i.e. carer/care worker (social care) or home help?
   4. Is there anyone else who helps you? E.g. Roles of people you live with/visits from other family members or friends – what do they help with?
   5. Do you have any help from aids? E.g. pill box, telecare pill dispenser? How did you find out about these aids i.e. pharmacist, GP, nurse?
4. Does your GP or someone at the practice do an annual dementia review with you where he/she talks to you and asks how things are going? (this would include a review of how you are doing, your health, your medication, what support you are getting etc.)
   1. probe if possible - If yes, what happens in them, who leads them, are medications discussed?
   2. have you ever had your medication reviewed? What happened?
5. Who helps with your medication (use term familiar) nowadays i.e. prescribing, delivering tablets (or other items), answering any queries related to your tablets?
   1. Do you talk to a pharmacist in a pharmacy or chemist shop/supermarket, or at your GP practice, or over the phone, about your medication? If yes move to next questions:
   2. What is helpful (or unhelpful?) e.g. booking system, appointment type i.e. remote/ face to face, ease of getting repeat prescription; changing a prescription?
   3. Easy to follow advice/information? (any cultural/language barriers, online/digital barriers?)
   4. Enough time/opportunity to talk through any problems/questions?
   5. Feel you are being listened to or not? (paying attention to you?)
   6. Being told about services/support you weren’t aware of before? E.g. pharmacist providing medications in Dosette boxes?
   7. Feelings of trust/ rapport/continuity of care
6. Have you started taking new medicines/tablets/drugs in the last 6 months?
   1. Who suggested this? Was it someone in your GP practice or someone else?
   2. Do your family / carer go with you to doctor’s appointments?
   3. Did they WHO? explain about your tablets and why you were starting new ones?
   4. Any help with swallowing tablets or taking them? E.g. liquid, patches, help with dissolving or cutting tablets?
7. Have any medicines/tablets/drugs been stopped or taken off?
   1. Who suggested this? Was it someone in your practice or someone else?
   2. Do your family / carer go with you to doctor’s appointments?
   3. Did they explain why you were stopping or changing/starting new ones? Was this helpful?
8. **Not a question but explanation for subsequent questions.** You have told me you get your medicines/tablets (what interviewee has mentioned) from the GP/Chemist (or what has been said, or if family carer or someone else gets them). Sometimes a chemist/pharmacist works in a GP surgery and might be able to help with your (medication/tablets), such as prescribing or taking you off tablets you might not need any more.
9. **Only ask if appropriate** - What might improve your experience with a pharmacist or *person who helps with medication*? (if at all)?
   1. What could they do/offer differently /that they aren’t currently?
   2. Understanding your circumstances (hearing problems, mobility problems, language barriers, swallowing, memory problems, challenges at home etc.)
   3. More flexibility i.e. appointment times, home visits, length of appointment...
   4. Improved accessibility (e.g., direct phone number/practice switchboard/email, face-to-face vs. remote access?)
   5. Clearer communication during or after the appointment?
   6. Hearing about additional support or services available to you?
   7. How might they support you and (your carer/family) better?
10. So just to sum up (if not covered) what support do people living with dementia need from pharmacists?
    1. How could they support others that are helping/supporting you (i.e., carer/family member)?
    2. Help with medication?
    3. Help with referrals to other services?
    4. A direct number to call when needed?

End of interview follow up questions

Before finishing and say goodbye – summarise their contribution to research and give thanks.

- Do you feel the length of our conversation was okay?
- Did it cause you any distress?
- Did you find talking about this subject/topic helpful? If not, what might have helped me.
- Is there anything else you would like to add/mention?
- Would you like me to leave /send you details of your local dementia support group or national Alzheimer’s Society or Dementia UK Admiral Nursing service help line if you don’t already have them?
