# Supplementary material for: Understanding the perspectives of people with dementia and family carers about clinical pharmacists in primary care: A qualitative study
Source: PLoS One. 2025 Aug 13;20(8):e0330028. doi: 10.1371/journal.pone.0330028 (PMC12348983; doi:10.1371/journal.pone.0330028)
Supplement: Attachment — Submitted filename: Reviewer responses v2_ND AB.docx [file pone.0330028.s003.docx]

| Reviewer’s comments | Author response |
| --- | --- |
| Methods 1. Page 9, line 232: Is "clinician" the correct term here? The revision is unclear—please consider clarifying or rephrasing. | Thank you for highlighting this, this has been changed to enhance clarity. |
| 2. How was the interview guide developed? Was it based on literature, and did you use an inductive or deductive approach? Was a pilot interview conducted? I could not locate the interview schedule in the version I received—possibly a formatting issue? | Lines 245-250 explain how the interview guide was developed. The Interview Schedule was also added as Appendix 1. |
| 3. Please indicate how many participants were interviewed via phone, video, or in person. Remote interviews with people living with dementia can be challenging. If in-person interviews were not feasible, please explain and discuss this as a possible limitation, ideally supported by relevant literature. | This has been added into the results section lines 298-301. It has also been added as a limitation lines 659-662. |
| Results 1. Some participants (how many?) had no prior contact with clinical pharmacists. This could be also mentioned later as a limitation, although it may have helped address recruitment challenges. | This has been added into the results section 304-305. However, we do not see this to be a limitation as the majority of participants had experience of a clinical pharmacist, and we were able to explore from the additional participants what support they *need* and how a clinical pharmacist *could* help. |
| 2. I think about reordering the themes: starting with the current Themes 1 and 3 would better reflect your research questions. Theme 2, based on a broader perspective, might fit better at the end. | Thank you for your suggestion, the themes have been reordered. |
| 3. Is it standard in your analysis approach to include brief descriptions of sub-themes in the main theme titles? From my experience, this is not always necessary if sub-themes are described in the text. | This was added in our previous revision in response to reviewer feedback, which we think provides greater clarity and guidance for the reader. . |
| Discussion 1. I suggest focusing strongly on Themes 1 and 3 in the discussion. Theme 2 appears more speculative and might be best addressed at the end. | Thank you, the discussion has been adapted to reflect this. We have also added in additional research to further support themes (1) and (2). |
